# Voxel-based mapping of postoperative epilepsy risk in glioma patients: supplementary motor area and limbic system correlations

**DOI:** 10.3389/fneur.2025.1556286

**Published:** 2026-01-05

**Authors:** Nzoyoum Kuetche Mandela William, Lei Wang, Liu Xianzhi

**Affiliations:** 1 First Affiliated Hospital of Zhengzhou University, Zhengzhou, China; 2 Guiqian International General Hospital, Guiyang, China

**Keywords:** glioma, epilepsy, VLSM, limbic system, frontal lobe

## Abstract

**Objective:**

This study aims to explore the correlation between glioma location in the limbic system and the risk of secondary epilepsy.

**Methods:**

This retrospective study included 170 cases of lower-grade gliomas treated with initial surgery from July 2007 to July 2019, sourced from the Chinese Glioma Genome Atlas (CGGA) database (http://www.cgga.org.cn). Patients were categorized into epilepsy and non-epilepsy groups based on postoperative symptoms. Imaging data were obtained from the Imaging Center of Beijing Tiantan Hospital, and postoperative epilepsy episodes were collected through follow-up. T2-weighted (T2WI) DICOM raw image data were converted into NII images, and tumor-susceptible regions were delineated using MRIcro software. Standardized T2WI gliomas and regions of interest (ROI) were analyzed using voxel-based lesion-symptom mapping (VLSM) software (https://crl.ucsd.edu/?). Overlapping ROIs were mapped for each patient. For voxel analysis in epilepsy-susceptible regions, the voxel with the highest *t*-value was defined as the peak voxel (PV). If the ROI overlapped with this voxel, the glioma was considered to confer a higher risk of epilepsy at that location. Statistical analysis was conducted using SPSS to compare glioma involvement in limbic system regions identified in imaging reports with patients’ postoperative epilepsy history.

**Results:**

Voxels significantly associated with tumor-related epilepsy were primarily located in the medial frontal lobe’s supplementary motor area of the left hemisphere. The peak voxel at this location was *X* = 88, *Y* = 155, *Z* = 134 (*t*_max_ = 4.69, *p* = 0.041), indicating the highest correlation with tumor-related epilepsy. Conversely, voxels less sensitive to epilepsy were mainly in the upper anterior cingulate gyrus. The peak voxel for this region was *X* = 91, *Y* = 166, *Z* = 79 (*t*_max_ = 3.70, *p* = 0.857), indicating the lowest correlation with tumor-related epilepsy.

**Conclusion:**

Epilepsy-susceptible regions of tumor-related epilepsy are located in the supplementary motor area of the medial frontal lobe in the left hemisphere. Regions not susceptible to epilepsy could primarily be in the anterior upper cingulate gyrus. Gliomas involving the anterior cingulate gyrus in the limbic system are associated with a lower postoperative risk of tumor-related epilepsy.

## 1 Introduction

Gliomas are the most common primary brain tumors (1), and epilepsy is one of the most frequent postoperative symptoms associated with gliomas (2–5). Histopathological studies suggest that tumor-related epilepsy is linked to changes in the tumor microenvironment, such as elevated concentrations of excitatory neurotransmitters like glutamate, which can promote seizure activity (6, 7). Additionally, tumor-related epilepsy is more commonly observed in lower-grade gliomas (5, 8, 9). The occurrence of preoperative tumor-related epilepsy has been found to correlate with tumor location, with tumors involving the temporal or frontal lobes being more prone to inducing seizures (9–11).

For postoperative tumor-related epilepsy, a meta-analysis revealed that favorable outcomes are associated with factors such as patient age over 45 years, focal seizures, complete tumor resection, and a preoperative epilepsy duration of less than 1 year. However, no significant correlation was found between tumors involving the temporal lobe and the risk of postoperative epilepsy (12). Other studies, however, have identified supratentorial tumors involving the temporal lobe, parietal lobe, and thalamus as independent risk factors for postoperative epilepsy (13). Thus, the relationship between tumor-involved regions and postoperative tumor-related epilepsy remains unclear.

Voxel-based lesion-symptom mapping (VLSM) enables precise voxel-level analysis, facilitating the identification of correlations between lesion location and specific tumor-related manifestations (14–16). VLSM has been widely applied in glioma-related research, revealing associations between tumor location and various aspects such as cognitive function (17–24), molecular pathology (25–30), genomics (31, 32), and prognosis (33–35). Regarding tumor-related epilepsy, previous studies have primarily focused on preoperative epilepsy. VLSM studies on glioblastoma have shown that preoperative epilepsy correlates with lesions in the superior and posterior frontal lobes, insula, and superior temporal lobe, while postoperative epilepsy is associated with the superior frontal gyrus, supplementary motor area, medial frontal region, anterior superior corona radiata, inferior medial occipital region, and caudate nucleus (33, 36).

In the limbic system, tumors involving the cingulate gyrus are associated with tumor-related epilepsy, whether preoperative or postoperative, while postoperative epilepsy is linked to the body of the corpus callosum. In our previous research using VLSM, preoperative tumor-related epilepsy in lower-grade gliomas was found to be associated with lesions in the left premotor area, part of which includes the cingulate gyrus (37). However, quantitative studies investigating the relationship between postoperative tumor-related epilepsy and tumor-involved regions in lower-grade gliomas (WHO grades II and III) remain limited.

To address this gap, this study retrospectively analyzes MRI scans of patients with lower-grade gliomas, utilizing VLSM technology to map voxel-level correlations between postoperative tumor-related epilepsy and non-epileptic outcomes. By elucidating the relationship between tumor location and epilepsy occurrence, this study aims to inform epilepsy management strategies for patients with lower-grade gliomas.

## 2 Materials and methods

### 2.1 Study participants

This study included 170 patients with lower-grade gliomas treated with initial surgery between July 2007 and July 2019. The patients were selected from the Chinese Glioma Genome Atlas (CGGA) database^1^ and the external set from the First Affiliated Hospital of Zhengzhou University (38). Inclusion criteria were as follows:

1. Pathological confirmation of lower-grade glioma (WHO grade II or III) according to the 2016 WHO classification of central nervous system tumors (39).
2. Preoperative magnetic resonance imaging (MRI) with at least T2-weighted imaging (T2WI) sequences available.
3. Patients aged 18 years or older.
4. No prior radiotherapy, chemotherapy, biopsy, or other invasive procedures or adjuvant treatments before surgery.
5. Availability of complete clinical data.

The study was approved by the Ethics Committee of Beijing Tiantan Hospital and adhered to the principles of the Helsinki Declaration. Informed consent was waived, as patients consented to the use of their clinical data for medical research at the time of admission.

### 2.2 Magnetic resonance imaging and preprocessing

All 170 included patients underwent preoperative MRI examinations performed on a Siemens MAGNETOM Prisma 3T MR scanner (Siemens Healthcare, Erlangen, Germany) with an external set taken from the First Affiliated Hospital of Zhengzhou University. The MRI protocol and parameters included T2-weighted imaging (T2WI) sequences, with the following acquisition parameters:

- Axial acquisition.
- Repetition time (TR): 5,800 ms.
- Echo time (TE): 10 ms.
- Flip angle: 150 degrees.
- Number of slices: 24.
- Field of view: 240 × 188 mm^2^.
- Voxel size: 0.6 × 0.6 × 5 mm^3^.
- Matrix: 384 × 300.

The raw MRI data were saved in DICOM (Digital Imaging and Communications in Medicine) format. To facilitate image preprocessing, the files were converted to NIFTI (Neuroimaging Informatics Technology Initiative, “.nii”) format using the DICOM-to-NIFTI conversion tool in MRICRON software (https://www.nitrc.org/projects/mricron, McCausland Center for Brain Imaging, University of South Carolina). The converted files were indexed and stored in a database as backup data for subsequent analyses.

To delineate the tumor location and signal intensity, tumor-related information was extracted from the MRI scans. Tumor segmentation was performed based on the T2WI sequence to define regions of interest (ROI). Areas with abnormal signal intensities on T2WI were considered indicative of lower-grade gliomas (40–43). Tumor ROIs were segmented using MRIcro software.^2^ The ROI boundaries for each patient were independently delineated by two experienced neurosurgeons (with over 5 years of neuroimaging experience). Consistency analysis was conducted, and if the difference between their delineated ROIs exceeded 5%, a senior radiologist (with more than 20 years of neuroimaging experience) reviewed the images and defined the final tumor boundaries. All three physicians were blinded to the clinical data of the lower-grade glioma patients.

The T2WI images and segmented ROIs were then registered to the standard brain space (Montreal Neurological Institute, MNI-152 space). The registration process employed the standard nonlinear spatial normalization algorithm implemented in SPM8.^3^ Each patient’s images were resampled to 1 mm × 1 mm × 1 mm resolution for higher spatial accuracy (44–47).

### 2.3 Assessment of tumor-related epilepsy

Postoperative epilepsy was assessed through annual telephone follow-ups, starting 1 year after surgery. Patients and their family members provided detailed accounts of postoperative epilepsy, including the presence of seizures, the timing of seizures, seizure types, and postoperative antiepileptic treatments. Seizure types were classified based on the 2017 International League Against Epilepsy (ILAE) classification (48), categorizing seizures by their origin (focal onset or focal to bilateral tonic-clonic), level of awareness (impaired or retained awareness), motor or non-motor features, and more specific seizure subtypes.

### 2.4 Voxel-based lesion-symptom mapping

Standardized lower-grade glioma T2WI and ROI images were analyzed using voxel-based lesion-symptom mapping (VLSM) software^4^ to map the overlapping ROI regions across patients.

VLSM analysis employs a General Linear Model (GLM), which is widely used in medical statistics and can be adapted for various analyses, including ANOVA, regression, ANCOVA, and multilevel regression. The general relationship between the dependent variable *Y* and independent variables *X* in a GLM is expressed as follows in Equation 1:


Y=βX+e (1)

where:

- *Y* is the vector of observed dependent variable values.
- *X* is the design matrix of independent variables.
- *β* is the vector of regression coefficients.
- *e* is the vector of independent random errors.

In this study:

- *Y*: indicates tumor involvement at a voxel (1 = involved, 0 = not involved).
- *X*: symptom matrix, where *X*1 is a constant symptom (1 = seizure, 0 = seizure-free), and *X*2, *X*3, … represent variables like gender and age etc.

The GLM computes the coefficient *β*1 for *X*1 and its corresponding *t*-value to test its significance (via *p*-value). A voxel is retained if its *t*-value exceeds a threshold determined through permutation testing (permutation testing, *n* = 1,000) (49). The threshold corresponds to the top 5% of *t*-values in the permutation distribution (*α* = 0.05, power >0.8).

The final seizure-prone region is defined by the retained voxels. Conversely, if seizure-free status is defined as *X*1 = 1 and seizure as *X*1 = 0, the analysis identifies the seizure-free sensitive region.

The voxel with the highest *t*-value in a sensitive region is defined as the peak voxel (PV). If an ROI involves the PV, the patient has a higher risk of experiencing the corresponding symptom.

### 2.5 Statistical analysis

All VLSM-related statistical analyses were conducted using MATLAB 2014a (The MathWorks Inc., MA, United States), while clinical variables were analyzed using R (version 3.6.3, R Foundation for Statistical Computing, Vienna, Austria). A *p*-value <0.05 was considered statistically significant, and all tests were two-sided.

Categorical variables were described as proportions, while continuous variables were evaluated for normality using the Shapiro–Wilk test (S–W test).

- For normally distributed variables (*p* > 0.05), results were presented as mean ± standard deviation (SD).
- For non-normally distributed variables (*p* < 0.05), results were presented as median ± interquartile range (IQR).

Statistical tests included

- Categorical variables: Pearson’s chi-square test or Fisher’s exact test, depending on cell frequencies (chi-square if theoretical frequencies ≥5, otherwise Fisher’s exact test).
- Continuous variables:
  - For normally distributed variables: unpaired *t*-test or analysis of variance (ANOVA).
  - For non-normally distributed variables: rank-sum tests such as Kruskal–Wallis (K–W test) or Mann–Whitney (M–W test).

## 3 Results

### 3.1 Clinical characteristics of patients

The primary clinical and pathological characteristics of the 170 patients are summarized in Table 1. Among the cohort, 119 patients (70%) experienced postoperative epilepsy, while 51 patients (30%) remained seizure-free.

**Table 1:** Association of demographic factors and brain region involvement with postoperative seizure occurrence (*N* = 170).

Clinical information	Postoperative seizures	No postoperative seizures	Total	*p*-value
Total number (%)	119 (70)	51 (30)	170	—
Gender distribution (male, %)	64 (53.78)	33 (64.71)	97 (57.06)	0.25
Age (mean ± SD)	40.03 ± 9.92	39.88 ± 11.35	—	0.934
Lobar involvement				
Parietal lobe	15	10	25	0.001
Frontal lobe	44	86	130	0.041
Temporal lobe	19	55	74	0.278
Insula	14	39	53	0.489
Occipital lobe	1	1	2	0.552
Limbic system				
Hippocampus	0	7	7	0.024
Amygdala	0	2	2	0.231
Thalamus	1	5	6	0.444
Corpus callosum	5	16	21	0.500
Cingulate gyrus	2	4	6	0.857
Mammillary body	0	0	0	—
Fornix	0	0	0	—
Hypothalamus	0	0	0	—

Among the 170 patients, 119 (53.78%) experienced postoperative epilepsy. Statistical analysis showed that age and gender had no significant influence on postoperative seizures (*p* > 0.05).

- Lobar involvement: seizures were significantly more associated with parietal lobe involvement (*p* = 0.001) and less frequent with frontal lobe involvement (*p* = 0.041 and *p* = 0.041). No significant associations were observed for the temporal, insular, or occipital lobes (*p* > 0.05).
- Limbic system involvement: hippocampal involvement was significantly associated with postoperative seizures (*p* = 0.024), while other regions, such as the amygdala, thalamus, corpus callosum, and cingulate gyrus, showed no significant associations (*p* > 0.05).

Completely excluding the preoperative seizures from the no postoperative seizures group, that is the non-epilepsy group we had in all 44 patients (Supplementary Table 1).

### 3.2 Relationship between glioma location and risk of secondary epilepsy

**Figure 1:**
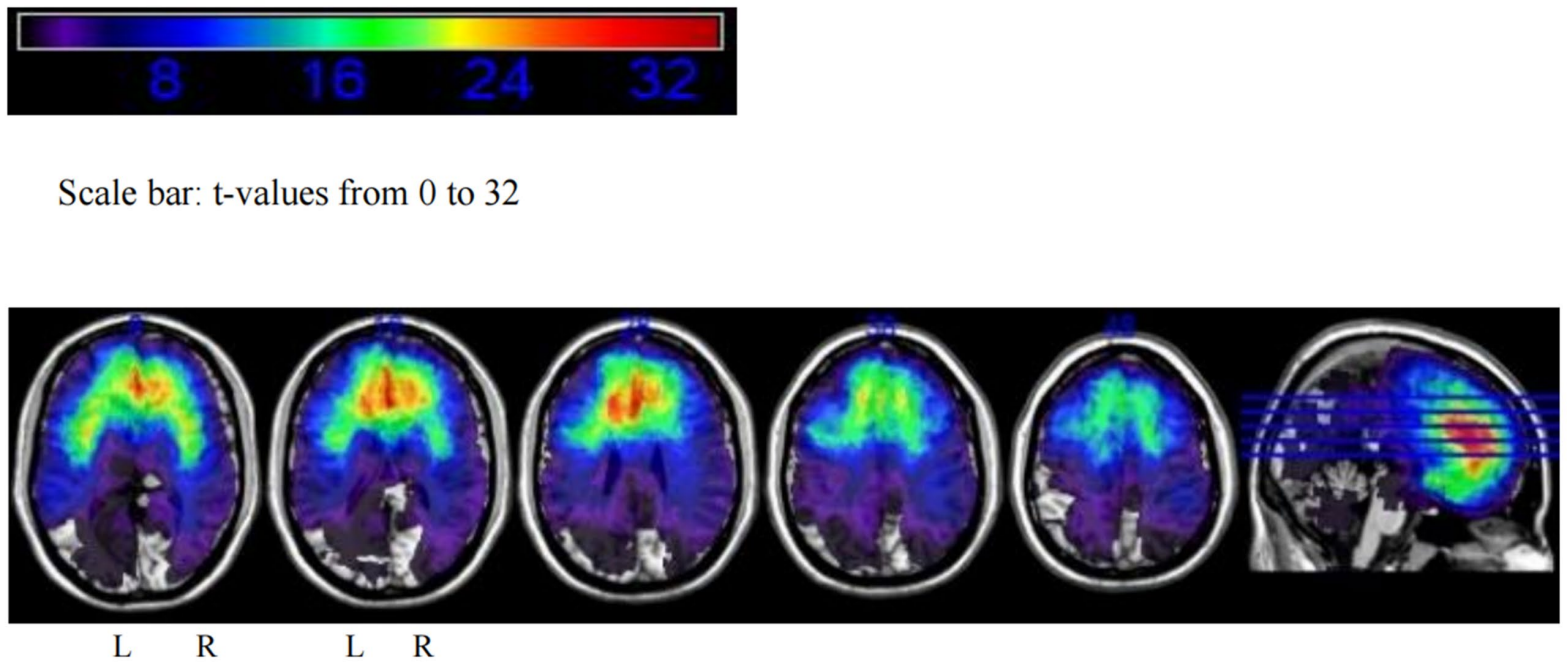
Tumor overlay map of glioma locations in 170 patients.

Based on the tumor locations of 170 patients, an overlay map was created to summarize all lesions in these glioma patients (Figure 1). This map shows that many brain regions, including the bilateral frontal lobes, temporal lobes, and insular cortices, exhibit significant tumor overlap.

Additionally, to identify voxel distributions with sufficient statistical power, a power map based on *p*-values was constructed to detect voxels with adequate reliability in the VLSM analysis (*p* < 0.05). In this study, only results within regions with high reliability (>0.8) were included in the final analysis.

**Figure 2:**
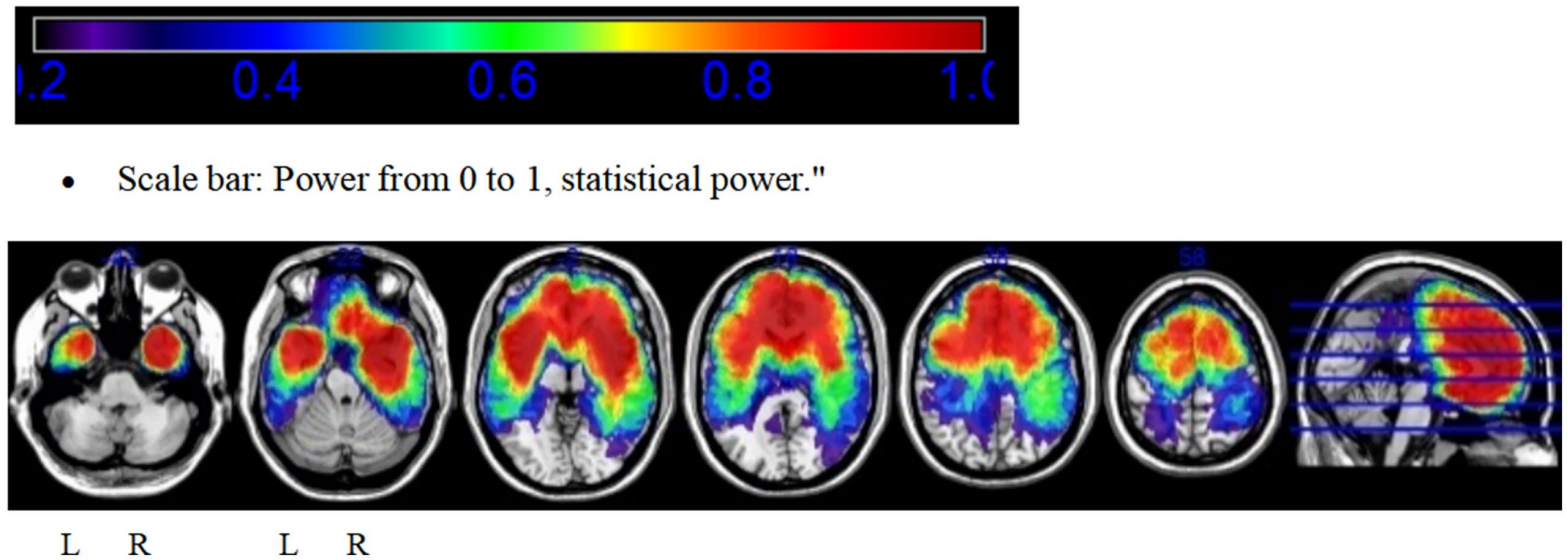
Power map highlighting brain regions with sufficient statistical power for voxel-based lesion-symptom mapping (*p* < 0.05, power >0.8).

The power map demonstrates that the majority of regions in both hemispheres provide highly reliable statistical analyses (Figure 2).

**Figure 3:**
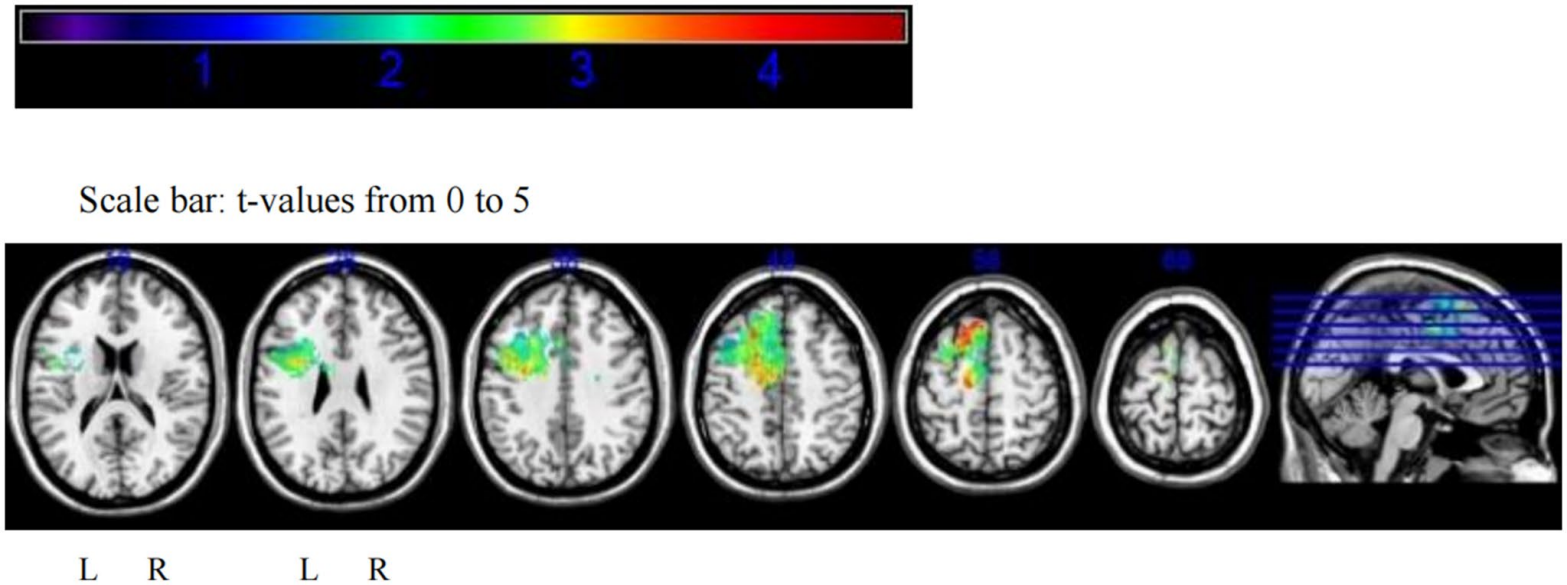
VLSM analysis highlights the medial supplementary motor area of the left frontal lobe as the region most strongly associated with tumor-related epilepsy.

**Figure 4:**
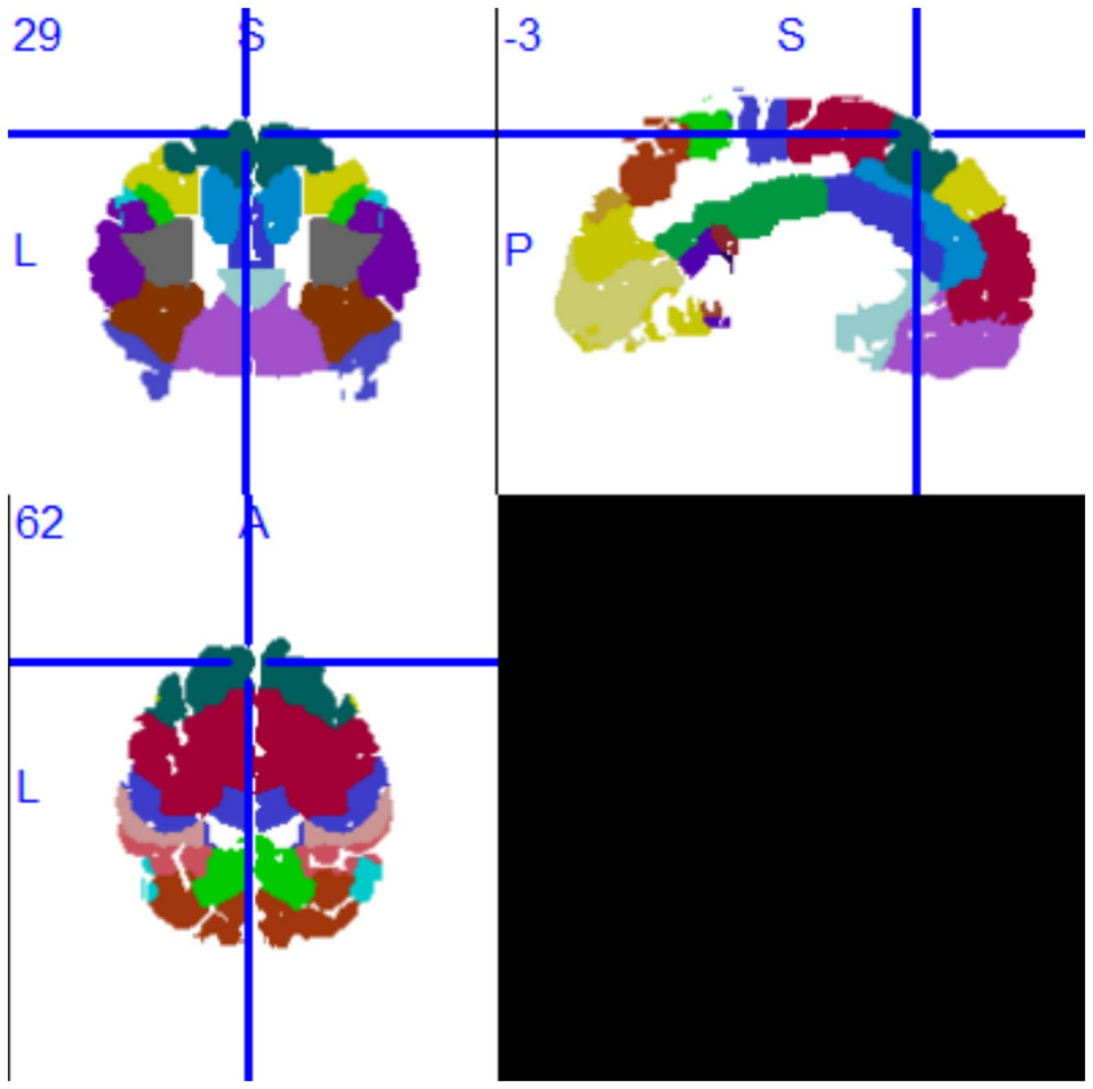
MRIcron Brodmann Area 8.

The VLSM analysis of this study, performed using MATLAB, identified the peak voxel (PV) coordinates as *X* = 88, *Y* = 155, *Z* = 134, with a *t*-value of *t*_max_ = 4.69. Mapping these results onto the MNI brain structure template and analyzing with MRIcron software revealed that the voxels significantly associated with tumor-related epilepsy were primarily located in Brodmann Area 8, specifically in the supplementary motor area (SMA) of the medial left frontal lobe. Based on this finding, we conclude that the SMA of the medial left frontal lobe demonstrates the strongest association with tumor-related epilepsy (Figures 3, 4).

**Figure 5:**
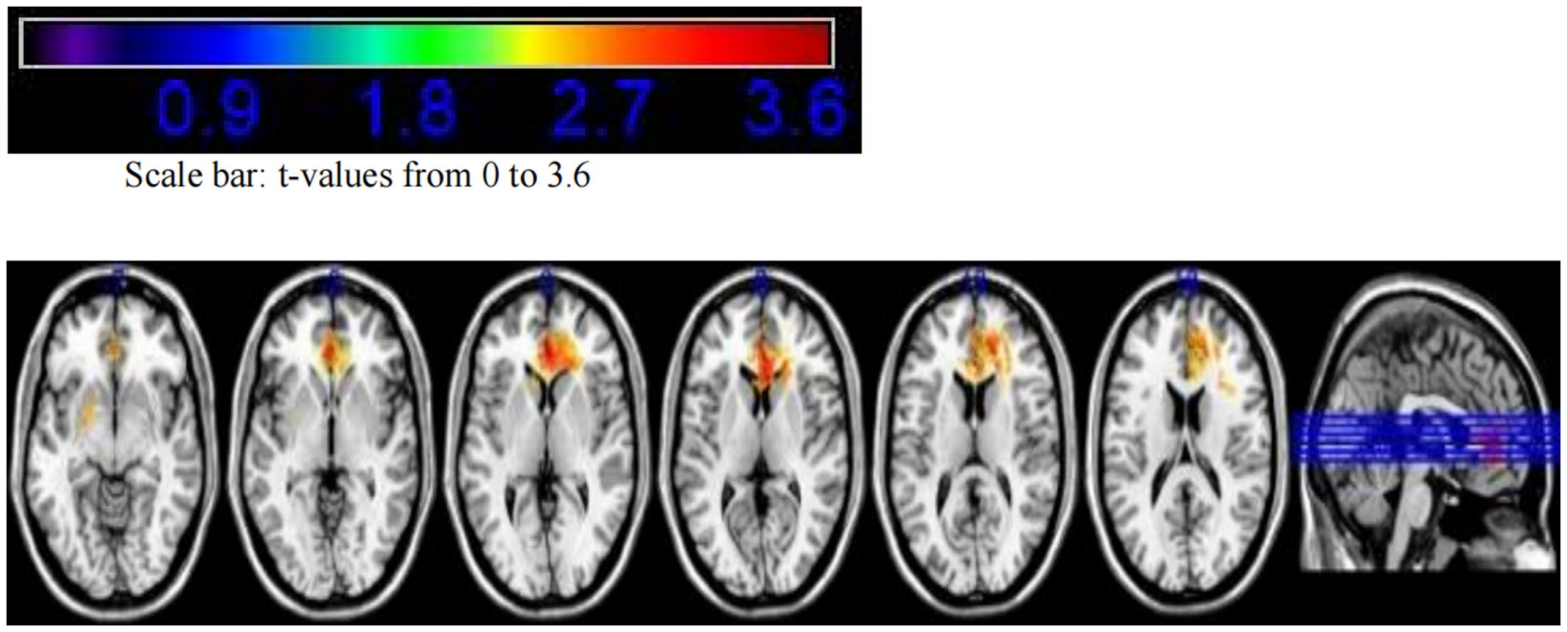
VLSM analysis indicates that the superior anterior cingulate cortex has the lowest association with tumor-related epilepsy.

**Figure 6:**
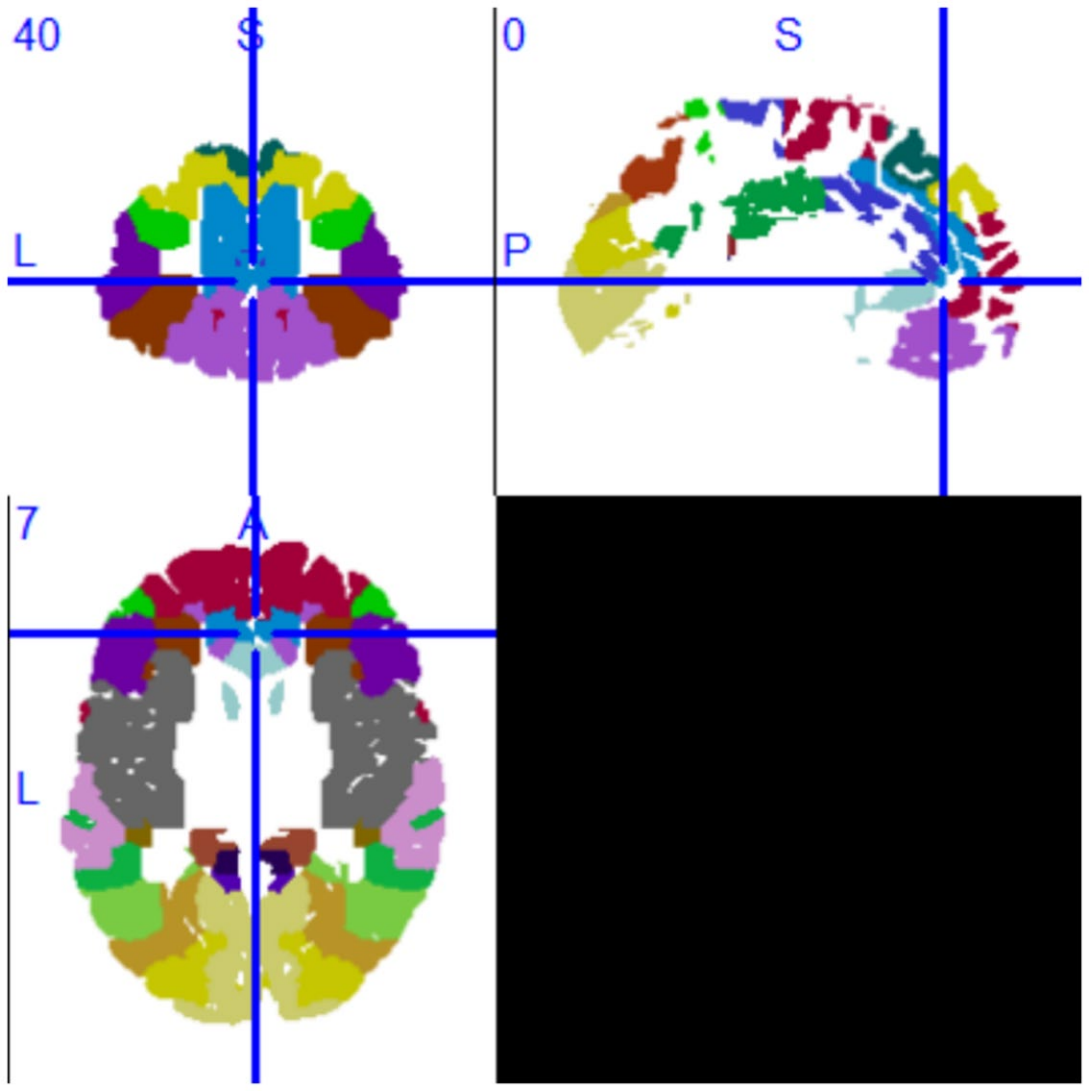
MRIcron Brodmann Area 32.

Additionally, we calculated the regions insensitive to tumor-related epilepsy. Following the VLSM analysis using MATLAB, the peak voxel (PV) coordinates for this region were identified as *X* = 91, *Y* = 166, *Z* = 79, with a *t*-value of *t*_max_ = 3.70, corresponding to MRIcron Brodmann Area 32. Mapping this voxel onto the MNI brain structure template revealed that the voxels insensitive to tumor-related epilepsy were primarily located in the superior anterior cingulate cortex. Based on this finding, we conclude that the superior anterior cingulate cortex has the lowest association with tumor-related epilepsy after surgery (see Figures 5,6).

### 3.3 Validation of epilepsy-susceptible regions identified by VLSM

Statistical analysis confirmed that the cingulate gyrus within the limbic system has the weakest correlation with postoperative epilepsy (*p* = 0.857). In contrast, the frontal lobe exhibits a certain degree of correlation. Additionally, compared to other brain lobes, the frontal lobe had the largest sample size in this study (*p* = 0.041) (Table 1).

## 4 Discussion

Tumor-related epilepsy is one of the most common symptoms following glioma surgery. This study aimed to determine the correlation between tumor-involved regions and postoperative tumor-related epilepsy at the voxel level and further explore whether low-grade gliomas involving the limbic system increase the risk of postoperative epilepsy. Retrospective analysis was conducted on 170 cases of low-grade gliomas (WHO grade II and III), employing VLSM (voxel-based lesion-symptom mapping) to identify epilepsy-prone regions. The epilepsy-prone area was found to be in the left medial frontal lobe’s supplementary motor area, while the non-epilepsy-prone region was primarily located in the anterior cingulate gyrus. Tumors involving the limbic system were associated with a lower risk of postoperative tumor-related epilepsy. These findings highlight the correlation between tumor-involved regions and postoperative epilepsy, offering valuable insights for predicting prognosis and developing anti-epileptic strategies for low-grade glioma patients. Statistical analysis revealed that the parietal and frontal lobes were more susceptible to postoperative epilepsy. Within the limbic system, gliomas involving the hippocampus showed a higher likelihood of causing epilepsy, likely due to its proximity to the temporal lobe.

### 4.1 Tumor-related epilepsy

Gliomas are the most common primary intracranial tumors (50). Epilepsy is one of the most frequent symptoms both preoperatively and postoperatively in glioma patients (51). In low-grade gliomas, preoperative tumor-related epilepsy occurs in approximately 65–90% of cases, with 70–90% of gliomas presenting with epilepsy as the initial symptom. Focal seizures progressing to bilateral tonic-clonic seizures account for approximately 67% of cases. In glioblastomas, epilepsy occurs in about 30–62% of patients, with two-thirds presenting with epilepsy as the initial symptom. Among these, 40% experience focal seizures progressing to bilateral tonic-clonic seizures, and 12% experience status epilepticus. Uncontrolled tumor-related epilepsy can severely impact patients’ quality of life and impose economic and emotional burdens on both patients and caregivers (52, 79). Conversely, preoperative tumor-related epilepsy is considered a favorable prognostic factor, as glioma patients with seizures generally have better outcomes (51).

The mechanism of tumor-related epilepsy is distinct from that of primary epilepsy. The epileptogenic focus is often located in the peritumoral cortex (53) rather than within the tumor core (80). Two primary mechanisms have been proposed: (1) Epileptic activity originates in peritumoral tissues due to mechanical compression, mass effect, local ischemia, hypoxia, and a reduction in pH, leading to the formation of epileptogenic foci (9, 53, 81, 82). The slow growth and infiltration of the tumor cause nerve conduction block in subcortical local and distant networks (83, 84). (2) Epileptic activity originates from the tumor itself, which secretes excitatory neurotransmitters such as glutamate, altering the microenvironment and disrupting the balance between excitatory and inhibitory mechanisms (9, 11, 54–58). Additionally, early postoperative epilepsy (within the first week) may be influenced by tumor location, histology, volume, edema, and postoperative complications (85–88). In high-grade gliomas, ischemia or hypoxia may contribute to early postoperative seizures (59, 60).

According to the 2016 WHO classification of central nervous system tumors (39), IDH mutations are one of the most critical molecular markers for glioma classification and prognosis. Several studies have identified a significant correlation between IDH mutations and preoperative tumor-related epilepsy (61–64). IDH mutations may contribute to postoperative epilepsy by producing D-2-hydroxyglutarate (D2HG), a structural analog of glutamate that mimics excitatory neurotransmitter activity at NMDA receptors, leading to hyperactive neuronal circuits and increased susceptibility to seizures (63). Exogenous D2HG also can increase the discharge rate of rat cortical cells (89–91). However, some studies suggest no significant association between IDH1 mutations and epilepsy in anaplastic gliomas (65). Another meta-analysis study found that in low-grade gliomas (WHO grade II), IDH mutation is associated with preoperative tumor-related epilepsy, with an odds ratio (OR) of 2.47 (95% confidence interval [CI], 1.70-3.57), however in higher-grade gliomas (WHO grades III-IV) there was no significant correlation (92).

Surgical resection, particularly removal of more than 90% of the tumor volume, is one of the most effective methods for controlling tumor-related epilepsy (11, 66–69). While some studies indicate that the extent of resection is unrelated to seizure control (93, 94), intraoperative electrocorticography (ECoG) may reduce postoperative epilepsy incidence (70, 71). Adjuvant treatments, including radiotherapy (96) and chemotherapy (95), have been shown to reduce seizure frequency by 50–60%, with 20–40% of patients achieving seizure freedom. The mechanisms may involve altering the microenvironment of epileptogenic foci or disrupting seizure-conducting pathways (72). Recurrent seizures after long-term seizure control in postoperative patients may be associated with tumor recurrence, particularly in glioblastoma (97).

A meta-analysis of postoperative epilepsy in glioma patients highlighted the association between postoperative epilepsy and factors such as age, preoperative seizure duration, extent of resection, and seizure type. Patients with focal seizures had a 32% higher risk of postoperative epilepsy, while those with generalized seizures had a 23% lower risk. Gross total resection increased the likelihood of seizure control by 47%. Patients aged ≥45 years and those with a preoperative seizure history of <1 year were more likely to achieve seizure control (12).

Evidence-based antiepileptic therapy is encouraged for managing tumor-related epilepsy (98–101). Levetiracetam is considered a first-line treatment due to its efficacy and safety profile (73). Lacosamide has also shown efficacy but is associated with side effects such as dizziness (74). This study underscores the importance of understanding the mechanisms, risk factors, and management strategies for tumor-related epilepsy, providing guidance for improving patient outcomes and quality of life (79).

### 4.2 Tumor location and tumor-related epilepsy

Low-grade gliomas involving the perilesional system, especially the insular lobe and frontal motor areas, are associated with tumor-related epilepsy (75). A meta-analysis including 16 studies with a total of 4,323 patients showed that tumor location is the strongest predictor of tumor-related epilepsy. Gliomas involving the occipital lobe were least likely to cause preoperative tumor-related epilepsy, while those involving the frontal lobe had the highest likelihood. In contrast, the parietal, occipital, and temporal lobes showed no significant correlation with tumor-related epilepsy (76). The mechanism behind the higher epilepsy risk associated with gliomas involving the frontal lobe remains unclear. On one hand, the frontal lobe is the largest among all brain lobes, and gliomas are most likely to form in this region, thus increasing the incidence of epilepsy related to gliomas in this area (77). On the other hand, the frontal lobe has widespread connections with surrounding brain regions, including the thalamus, basal ganglia, and brainstem. Abnormal discharges from the frontal lobe can spread to these areas, triggering corresponding seizures. Tumors in the frontal lobe primarily affect the cortex and superficial brain tissues, with minimal involvement of deep white matter fibers, which helps facilitate the spread of abnormal discharges (78).

In a VLSM study on glioblastomas, preoperative tumor-related epilepsy was found to be associated with the right superior, middle, and inferior temporal gyri, the right fusiform gyrus, the parahippocampal gyrus, the right anterior and posterior central gyri, the right middle frontal gyrus, the left posterior central gyrus, the left superior frontal gyrus, and the bilateral cingulate gyri. The voxel distribution related to epilepsy was extensive, with the largest voxel clusters found in the right anterior and posterior central gyri, the left posterior central gyrus, and the right temporal lobe and parahippocampal gyrus, indicating that tumors affecting these regions have the highest value for analyzing tumor-related epilepsy (33).

In our study, the epilepsy-prone area was found to be in the left medial frontal gyrus in the supplementary motor area, while the non-epilepsy-prone area was primarily in the anterior part of the cingulate gyrus. Therefore, tumors involving the limbic system have a lower risk of postoperative tumor-related epilepsy. Combining the findings from the above VLSM-based studies, the pre-motor area appears to be the most important epilepsy-sensitive area, likely due to the fact that motor seizures are more easily observed. Regarding the limbic system, the relationship with tumor-related epilepsy is contradictory. Tumors involving the parahippocampal gyrus and cingulate gyrus are associated with preoperative epilepsy in high-grade gliomas, and those involving the anterior medial part of the cingulate gyrus, the knee and body of the corpus callosum are related to postoperative epilepsy. However, lesions in the posterior hippocampus, parahippocampal gyrus, fornix, and amygdala in high-grade gliomas showed no association with postoperative epilepsy, and lesions in the left cingulate gyrus were linked to preoperative epilepsy in low-grade gliomas. Our study, however, found no association between cingulate gyrus involvement and postoperative seizures in low-grade gliomas. This suggests that further large-scale prospective studies, combined with video EEG for epilepsy classification might be needed to better differentiate the correlation between different locations in the limbic system and epilepsy.

### 4.3 Limitations

This study is a single-center retrospective study, and its findings require further validation through large-sample, multi-center studies. The follow-up of postoperative epilepsy symptoms was mainly conducted through phone follow-ups, where descriptions from patients and their families were used to determine the presence and specific types of postoperative seizures. The lack of auxiliary examination methods, such as video-EEG, means that the diagnosis and classification of seizures may be biased.

## 5 Conclusion

This retrospective study included 170 patients with low-grade gliomas (WHO grade II and III). Using the VLSM method, we identified the epilepsy-prone area for tumor-related epilepsy, located in the left medial frontal gyrus in the supplementary motor area. The non-epilepsy-prone area was primarily located in the anterior part of the cingulate gyrus. Therefore, tumors involving the limbic system (such as the cingulate gyrus, corpus callosum, hypothalamus, and amygdala) have a lower risk of postoperative tumor-related epilepsy. This study clarifies the correlation between tumor involvement and postoperative tumor-related epilepsy, thus providing helpful guidance for prognostic prediction and the development of antiepileptic strategies for low-grade gliomas after surgery.

## Data Availability

The datasets presented in this article are available from the first author upon reasonable request. Requests should be directed to NM, mandelawilliam@163.com.

## Footnotes

1
http://www.cgga.org.cn

2
www.mricro.com

3
http://www.fil.ion.ucl.ac.uk/spm/software/spm8

4
http://crl.ucsd.edu/vlsm
